# Supplementary material for: Quantitative dissection of the metastatic cascade at single colony resolution
Source: Res Sq. 2026 Sep 9:rs.3.rs-10965122. Preprint. [Version 1] doi: 10.21203/rs.3.rs-10965122/v1 (PMC13622843; doi:10.21203/rs.3.rs-10965122/v1)
Supplement: Supplementary file 1 [file NIHPPrs10965122v1-supplement-1.pdf]

Supplementary Figure 1. Establish the MOBA-seq pipeline.

- **a.** Schematic of the MOBA-seq construct design, including cloning of the barcoded insert downstream of the U6 promoter and incorporation of sgRNA oligo pools. PCR amplification using flanking restriction sites (XbaI, Xho1) enables efficient library assembly and integration of random barcodes in the bovine U6 promoter. Golden gate cloning enables sgRNA oligo library insertion into barcode vectors.
- **b.** Flow cytometry validation of Cas9 cutting efficiency in H82, RP48, and RP116 cells. Self-targeting GFP/sgGFP reporters are used to transduce Cas9$^+$ and Cas9$^-$ cells. Cas9 cutting efficiency is normalized by percentage of GFP$^-$ cells in transduced Cas9$^+$ cells.
- **c.** Distribution of total cell counts per barcode illustrating barcode complexity and evenness across the library. The red dashed line marks the 95th percentile threshold used to filter high-abundance barcodes prior to downstream analysis.
- **d.** Quantification of mCherry-labeled H82 seeding efficiency via different injection routes in liver and lung. Percentage of mCherry$^+$ H82 cells in liver and lung are measured 2 hours and 48 hours post-transplantation. Error bars represent mean ± s.d. IC: intra-cardiac; IV: intra-veinous; ISp: intra-splenic.
- **e.** Overview of the MOBA-seq library amplification workflow. Barcoded genomic DNA from target organ is subjected to MOBA 1st-round PCR (20 cycles), column-purified, diluted, and followed by an NGS-compatible 2nd-round PCR (15 cycles).
- **f.** Linear regression between relative NGS read counts and expected barcode frequency for spike-in cells across samples. R^2=0.98 demonstrated a robust reproducibility for colony size calculation.
- **g.** Stacked distribution of R^2 values from linear regression analyses used to calculate colony sizes for samples derived from bone marrow, liver, lung, and blood. The consistently high R^2 values across tissues indicate strong confidence and robustness in colony size quantification for diverse sample types.
- **h.** Flowchart summarizing the LETTUCE computational pipeline for MOBA-seq data analysis, including read merging, filtering, UMI correction, cutoff selection, barcode collapse, and final metastasis-associated statistics analysis.
- **i.** Histogram of the number of metastatic colonies detected per mouse across experiments in C57BL/6 and NSG backgrounds. Mean colony number per mouse is indicated by dashed blue lines.
- **j.** Proportion of single-mouse-seeding SCLC clones identified in C57BL/6 and NSG mice following transplantation of barcoded RP48 cells via tail vein injection. The majority of barcoded clones seeded into only one mouse, indicating minimal multi-seeding events from the same barcode and ensuring high clonal assignment fidelity.
- **k.** Genotype-specific metastatic seeding is reproducible across individual mice. Absolute numbers of liver metastatic seeding events associated with each sgRNA genotype were compared between two biological replicates. Metastatic seeding frequencies were highly concordant between mice (R^2 = 0.94), demonstrating reproducible genotype-specific effects on liver metastatic seeding.
- **l.** Reproducibility of metastatic seeding profiles across biological replicates. Heatmap showing pairwise R^2 values for genotype-specific metastatic seeding profiles across six individual mice. Pairwise correlations ranged from R^2 = 0.65 to 0.94, demonstrating consistent metastatic seeding patterns across independent biological replicates.
- **m.** High-sensitivity detection of circulating tumor cells in C57BL/6 and NSG mice. Numbers of unique circulating tumor cell (CTC) colonies detected per milliliter of blood (*Top*) and total CTC cell numbers per milliliter of blood (*Bottom*) are shown for individual mice. Dashed lines indicate the mean across mice. These data demonstrate robust detection of metastatic CTCs in peripheral blood.

Supplementary Figure 2. MOBA-seq quantifies tissue-specific metastatic effects of *Nfib*.

- **a.** Schematic of the MOBA-seq experimental design to quantify tissue-specific metastatic effects of *Nfib*. RP48 SCLC Cas9$^+$ cells were transduced with pooled barcoded lenti-sgRNAs targeting either Safe controls or *Nfib*, followed by intravenous injection into NSG mice. After metastatic colonization in liver, lung, and brain, tissues were collected at defined timepoints and processed for barcode-based metastatic clonal reconstruction and metastatic effects analysis.

b. Stacked bar plots showing genotype-specific metastatic cell numbers across liver and lung at serial timepoints. Each bar depicts the proportional contribution of metastatic SCLC clones within each sgRNA group. Loss of *Nfib* reduced clonal representation in both liver and lung over time.

c. Quantification of relative metastatic burden for sgSafe and *Nfib*-targeting clones over time in liver and lung. *Nfib* inactivation significantly reduces metastatic burden in liver and lung comparing to sgSafe control.

d. Quantification of relative metastatic seeding for *sgSafe* and *Nfib*-targeting clones in liver and lung. *Nfib* inactivation led to a sustained reduction in initial seeding efficiency in the liver and progressively decreased lung metastatic colony numbers toward sgSafe control levels over time.

e. Quantile analysis of relative fitness among liver and lung metastatic colonies. Loss of *Nfib* produced significant negative fitness effects in larger liver colonies (≥75th percentile) at 3 weeks, whereas no significant fitness changes were observed in lung colonies. FDR < 0.05 are indicated.

f. Density distributions of colony sizes for *sgSafe* and *sgNfib* clones in liver and lung. Vertical lines denote the modal colony size (PeakMode) for each genotype at the indicated timepoints. *Nfib* inactivation shifts the distribution toward markedly smaller colonies in the liver, whereas no detectable effect on colony-size distributions is observed in the lung.

g. MOBA-seq identifies cutoffs for dormant colony sizes and the valley mode between dormant and expanding colonies across liver, lung, and brain. The distribution of log2-transformed tumor cell number of the pooled tumors across liver, lung, and brain at the 3-week time point was plotted, and the dormant mode and valley mode from the pooled distribution was applied across all three tissues to determine their dormant populations. This qualitative framework allows for the unbiased calculation of dormancy across tissues, time points, and sgRNAs.

h. Gaussian mixture modeling (GMM) illustrating the fraction of dormant colonies in liver and lung for *sgSafe* and *Nfib*-targeting metastases. Unexpectedly, *Nfib* inactivation decreased the proportion of dormant colonies in the liver but not in the lung.

i. Comparison of genotype-specific SuperMet colonies across time in the lung. Left: Scatterplots display colony size for *sgSafe* and *Nfib*-targeting metastases in the lung. SuperMet colonies that detected in the blood were labeled in red. Right: Colony size distributions showing SuperMet colony size from *sgSafe* and *sgNfib* metastases. *Nfib* inactivation has no effect in SuperMet colony size in the lung.

Supplementary Figure 3. MOBA-seq identified known and novel SCLC metastasis regulators.

a. Summary plot of candidate genes included in the MOBA500 library. Circle size reflects mutation frequency in human SCLC patients, and point color denotes T-statistics derived from prior CRISPR screening in SCLC cell lines. The top 10 genes in each category are labeled.

b. MOBA-seq identifies tumor suppressors in SCLC lung and brain metastasis. Metastatic colonies from each genotype are ranked by their mean colony size, which is indicated by a red dot. The dashed line represents the mean colony size of all control colonies combined (sgInerts).

c. MOBA-seq identifies known and novel genetic regulators in SCLC metastasis in the lung and brain. Ranked plot of each genotype highlights top candidate suppressors (red, Log2FC>1, FDR<0.05) and drivers (blue, Log2FC<-1, FDR<0.05) of SCLC lung (left) and brain (right) metastatic burden. Grey error bars represent 95% confidence interval. sgRNAs that generate wild-type metastases are shown in green.

d. MOBA-seq identifies metastatic fitness landscape in whole mouse. Ranked plot of each genotype highlights top candidate suppressors (red, Log2FC>1, FDR<0.05) and drivers (blue, Log2FC<-1, FDR<0.05) of whole mouse metastatic burden. Grey error bars represent 95% confidence interval. sgRNAs that generate wild-type metastases are shown in green.

e. Comparison of mutation frequency in human SCLC tumors versus whole-mouse metastatic fitness derived from the MOBA-seq screen. Genes harboring high-frequency mutations in patients that also exhibit reduced metastatic fitness *in vivo* (red) represent putative metastasis suppressors with strong clinical relevance (e.g., *PTEN* and *CREBBP*).

f. Comparison of T-statistics from prior CRISPR screening in SCLC cell lines with whole-mouse

metastatic fitness from the MOBA-seq experiment. Genes showing strong negative or positive T-statistics and large metastatic fitness effects include known SCLC essential genes (e.g., *ASCL1*) as well as several newly identified metastasis-associated genes (red).

Supplementary Figure 4. Metastatic seeding is a reproducible and size-independent metric of metastasis.

a. Metastatic seeding is independent of pre-transplantation cell abundance. Absolute pre-transplantation cell number and relative liver metastatic seeding fold change were plotted for each sgRNA. Pre-transplantation cell abundance, which was used to normalize metastatic seeding and also reflects genotype-specific proliferation during in vitro culture, showed no correlation with metastatic seeding ($R^2 = 0.00$), indicating that the enhanced metastatic phenotype cannot be explained solely by differences in cell proliferation before transplantation.

b. Metastatic seeding is independent of peak-mode metastatic colony size. Relative liver metastatic seeding fold change was compared with relative peak-mode metastatic colony size for each sgRNA. The two measurements showed essentially no correlation ($R^2 = 0.00$), indicating that genotype-specific effects on metastatic seeding are distinct from effects on subsequent metastatic colony expansion.

c. Metastatic seeding is independent of metastatic colony size across the colony-size distribution. For each colony-size percentile, R^2 values were calculated across sgRNAs between relative liver metastatic seeding and relative metastatic colony size. Correlations remained minimal across the full range of colony-size percentiles, demonstrating that metastatic seeding and metastatic outgrowth represent largely independent phenotypes.

d. Genotype-specific metastatic seeding estimates are robust to metastatic colony-size thresholds. Heatmap showing relative liver metastatic seeding fold change for each sgRNA calculated using metastatic colony-size thresholds ranging from 1 to 1,000 cells. Genotype-specific seeding effects remained highly consistent across a broad range of colony-size cutoffs, demonstrating that metastatic seeding is a robust metric that is not dependent on the definition of a metastatic colony.

Supplementary Figure 5. MOBA-seq illustrates relationships between diverse metastatic metrics.

a. Metastatic seeding is the primary factor driving overall metastatic burden in the lung. Plot of genotype-specific metastatic seeding and metastatic burden for all genotypes revealed a good linear correlation (R2=0.68). Outlier suppressors from linear regression are marked in purple. Grey error bars represent 95% confidence interval.

b. Regression analyses evaluating the relationship between metastatic burden and multiple metastasis metrics in the lung, including 90th percentile colony size, peak mode, dormancy escape, and SuperMet percentage. Outlier from linear regression is marked in purple.

c. Regression analyses evaluating the relationship between metastatic burden and multiple metastasis metrics in the liver, including 90th percentile colony size, peak mode, dormancy escape, and SuperMet percentage. Outlier from linear regression is marked in purple.

d. Large metastatic colonies primarily determine outlier distance rather than metastatic burden. Metastatic colony size at different percentiles in the liver is used to model linear regression with outlier distance, metastatic burden, or metastatic seeding. The R^2 values for each regression are plotted against the corresponding percentile. Note that high correlations ($R^2 > 0.7$) are observed only at high percentiles ($>90^{th}$) when modeling with outlier distance.

e. Regression analyses evaluating the relationship between dormancy escape and multiple metastatic metrics in the liver and lung, including metastatic burden, 90th percentile colony size, and metastatic seeding. Dormancy escape shows a positive association with 90th colony size in the lung, whereas in the liver it is negatively correlated with 90th percentile colony size, highlighting tissue-specific differences in how clones exit dormancy and expand during metastatic progression.

Supplementary Figure 6. Metastatic suppressors and essential genes have distinct effects at different steps of the metastatic cascade.

- **a.** Ranked metastatic burden (log$_2$FC) for all genotypes in SCLC liver metastases. Inactivation of metastatic suppressors (red) increased overall metastatic burden, while inactivation of essential genes from the Rac-WRC-ARP2/3 pathway (blue) decrease overall metastatic burden. Error bars represent 95% confidence intervals.
- **b.** Ranked metastatic seeding (log$_2$FC) for all genotypes in SCLC liver metastases. Inactivation of most metastatic suppressors (red) increased metastatic seeding, with notable exceptions on *Tsc1* and *Tsc2*. In contrast, loss of essential genes within the Rac–WRC–ARP2/3 pathway (blue) consistently decreased metastatic seeding. Error bars indicate 95% confidence intervals.
- **c.** Ranked 90th percentile colony size (log$_2$FC) for all genotypes in SCLC liver metastases. Inactivation of most metastatic suppressors (red) increased colony size, with notable exceptions such as *Gata6* and *Csnk1a1*, which did not enhance clonal expansion. In contrast, loss of essential genes in the Rac–WRC–ARP2/3 pathway (blue) had minimal effects on 90th percentile colony size, with the exception of *Rac1*, which significantly reduced large-clone outgrowth. Error bars represent 95% confidence intervals.
- **d.** Ranked peak mode colony size (log$_2$FC) in SCLC liver metastases. Metastatic suppressors exhibit diverse effects on the exponential clonal expansion mode, reflecting heterogeneous control of metastatic growth. Loss of essential genes in the Rac–WRC–ARP2/3 pathway did not yield sufficient colonies to reliably calculate peak-mode colony size.
- **e.** Ranked dormancy escape percentage (log$_2$FC) in SCLC liver metastases. Metastatic suppressors display diverse effects on the ability of dormancy escape. Notably, inactivation of strong suppressors such as *Tsc2* and *Crebb*p markedly reduced dormancy escape in the liver. Loss of essential genes in the Rac–WRC–ARP2/3 pathway did not yield sufficient colonies to reliably calculate dormancy escape.
- **f.** Ranked SuperMet percentage (log$_2$FC) in SCLC liver metastases. Inactivation of most metastatic suppressors (red) increased SuperMet percentage. In contrast, loss of essential genes within the Rac–WRC–ARP2/3 pathway (blue) consistently decreased SuperMet percentage.

Supplementary Figure 7. MOBA-seq identifies genotype-specific metastasis effects in immune deficient mice.

- **a.** Genotype-ranked metastatic metrics in liver metastases from NSG mice, including metastatic burden, metastatic seeding, 90th percentile colony size, SuperMet percentage, peak mode size, and dormancy escape (all as in log$_2$FC relative to sgSafe).
- **b.** Genotype-ranked metastatic metrics in lung metastases from NSG mice, including metastatic burden, metastatic seeding, 90th percentile colony size, SuperMet percentage, peak mode size, and dormancy escape (all as in log$_2$FC relative to sgSafe).
- **c.** Genotype-ranked metastatic metrics in brain metastases from NSG mice, including metastatic burden, metastatic seeding, 90th percentile colony size, and SuperMet percentage (all as in log$_2$FC relative to sgSafe). Brain metastasis did not yield sufficient colonies to reliably calculate peak mode size, and dormancy escape.
- **d.** Targeting the Rac1-WRC-ARP2/3 pathway consistently affects metastatic burden and seeding but has little effect on colony size in both C57BL/6 and NSG mice. The error bars represent 95% confidence interval.
- **e.** MOBA-seq identified diverse patterns for metastasis suppression in both C57BL/6 and NSG mice. Radar plots stratify genetic suppressors by their effects on six metastatic metrics throughout the metastatic cascade.

Supplementary Figure 8. *Gpatch8* suppresses brain metastatic outgrowth in an immune-dependent manner.

- **a.** Inactivation of *Gpatch8* in RP48 cells increased brain metastatic burden (*left*), metastatic seeding (*middle*), and 90th-percentile metastatic colony size (*right*) in immunocompetent C57BL/6 mice relative to safe-targeting controls, supporting *Gpatch8* as a brain metastasis suppressor. Data points

outlined in black indicate statistically significant comparisons (FDR < 0.05).

b. Inactivation of *Gpatch8* in RP48 cells increased brain metastatic seeding (*middle*) but reduced overall brain metastatic burden (*left*) and 90th-percentile colony size (right) relative to safe-targeting controls in immunodeficient NSG mice. These divergent effects suggest that *Gpatch8* loss promotes initial brain seeding but does not enhance subsequent metastatic outgrowth in the absence of an intact immune system, supporting an immune-dependent role for Gpatch8 in suppressing brain metastatic expansion. Data points outlined in black indicate statistically significant comparisons (FDR < 0.05).

Supplementary Figure 9. Genotype-specific regulation of cross-tissue SuperMet dissemination.

a. Heatmaps showing cross-tissue dissemination of shared metastatic colonies in immunodeficient NSG mice. For each sgRNA genotype, the percentage of colonies originating in each tissue that were also detected in each destination tissue was calculated. Colors indicate the log2 fold change relative to control guides. Inactivation of *Crebbp*, *Tsc1*, and *Tsc2* increased cross-tissue dissemination of SuperMets, whereas loss of *Kctd10* and *Zeb1* reduced dissemination across tissues.

b. Heatmaps showing cross-tissue dissemination of shared metastatic colonies in immunocompetent C57BL/6 mice. For each sgRNA genotype, the percentage of colonies originating in each tissue that were also detected in each destination tissue was calculated. Colors indicate the log2 fold change relative to control guides. Inactivation of *Crebbp*, *Gpatch8*, and *Pten* increased dissemination of SuperMets between specific tissue pairs.

Supplementary Figure 10. Recipient sex modulates immune surveillance of SCLC metastasis.

a. Schematic of the MOBA-seq experimental design used to evaluate sex-specific metastatic phenotypes. Cas9$^+$ SCLC cells (RP48 and RP116) transduced with barcoded lenti-sgRNAs were injected intravenously into male or female immune-deficient NSG or immune-competent C57BL/6 mice (N = 5 per sex per mouse genotype). Following metastatic colonization in liver, lung, and brain, barcode-resolved clonal analysis was performed to quantify genotype-specific metastasis behavior.

b. RP48 and RP116 cells exhibited distinct metastatic growth phenotypes, whereas female recipient mice showed stronger suppression of metastatic seeding. Syngeneic SCLC cells of male (RP48) and female (RP116) origin were transplanted into both male and female recipient mice with immune proficient (C57BL/6) and deficient (NSG) backgrounds. **Left**: Barcoded liver metastatic colonies from five mice are plotted, with each point representing one colony. Color indicates the sex of mice, and shape indicates the sex of cancer cells. Note that male cells grow better, and female mice have a stronger immune response. **Right**: Metastatic colony size distribution of each mouse-cell sex combinations. Note that female but not male SCLC cells are more sensitive to immune suppressions evidenced by a decreased clonal expansion peak mode.

c. Heatmaps of genotype-specific metastatic burden ($\log_2$FC) in the liver. Each row corresponds to an sgRNA-defined genotype, and each column represents an individual mouse annotated by sex and immune background. Strong sex-dependent differences in metastatic suppression emerge, particularly in female C57BL/6 mice. Color bars indicate gene categories. Top panel: male-derived RP48 cells; bottom panel: female-derived RP116 cells.

d. Sex- and immune-stratified significance matrices for genotype-specific metastatic burden, metastatic seeding, dormancy escape, and percentile colony sizes (90th, 50th, and 20th) in liver and lung. Each square represents the measured difference in a genotype-specific metastatic metric for a given sex- or immune-state comparison, with significant associations (*FDR* < 0.05) indicated by a star within the box. Top panel: liver metastasis; bottom panel: lung metastasis.

Supplementary Figure 11. *Crebbp* is a potent metastasis suppressor.

a. *Crebbp* inactivation increases SCLC metastatic burden at three weeks. Bar plot showing the relative proportions of metastatic tumor cells by their genotype. Shown is the representation of SCLC genotypes recovered from liver, lung, brain, and bone marrow (BM) tissues of both NSG and C57BL/6 mice after three weeks of growth, compared with the pre-transplantation sample. Three *sgCrebbp* genotypes are

highlighted.

b. *Crebbp* inactivation increases SCLC metastatic seeding across multiple tissues. Metastatic colonies recovered from liver, lung, brain, and bone marrow at three weeks are shown, with each dot representing an individual barcode-defined colony and dot size proportional to colony cell number. Total colony counts and mean colony sizes are indicated for *sgNT3* and *Crebbp_sg2*, which exhibited comparable cell numbers in the pre-transplantation pool. Across all organs and timepoints, *Crebbp* inactivation consistently increased the number and size of metastatic colonies relative to controls.

c-h. *Crebbp* inactivation promotes liver metastasis in both RP116 and RP48 models across immune backgrounds. In RP116 cells (**c-e**), *Crebbp* inactivation increased relative metastatic burden (**c**), metastatic seeding (**d**), and 90th-percentile colony size (**e**) in the liver of both NSG and C57BL/6 mice compared with control sgRNAs. In RP48 cells (**f-h**), Crebbp inactivation increased relative metastatic burden (**f**) and metastatic seeding (**g**) in the liver in both NSG and C57BL/6 mice, whereas it increased 90th-percentile colony size (**h**) in NSG mice but not in C57BL/6 mice. For each time point, values are plotted as fold change relative to control sgRNAs. Data points outlined in black indicate statistically significant comparisons (FDR < 0.05).

Supplementary Figure 12. Crebbp is a shared metastasis seeding suppressor across multiple tissues.

a-d. Comparison of genotype-specific RP48 cell metastatic seeding fold changes between C57BL/6 and NSG mice in the liver (**a**), lung (**b**), brain (**c**), and bone marrow (**d**). Dashed lines indicate the wild-type reference level. Genotypes that significantly increased metastatic seeding in both immune backgrounds are highlighted as shared metastasis suppressors, whereas genotypes that significantly decreased metastatic seeding in both backgrounds are highlighted as shared metastatic drivers. *Crebbp* loss consistently increased metastatic seeding across multiple tissues, including liver, lung, brain, and bone marrow, identifying *Crebbp* as a broad metastasis suppressor whose effect is conserved across distinct metastatic tissue and immune contexts.

Supplementary Figure 13. cNMF analysis reveals that Crebbp loss shifts SCLC cell-state programs toward an SCLC-I-like state.

a. Selection of the optimal number of cNMF components based on solution stability and reconstruction error across the indicated numbers of components. Four components were selected for downstream analysis.

b. Gene expression programs (GEPs) identified by cNMF. Dot size and color indicate the relative contribution of individual genes to each program, highlighting distinct transcriptional features associated with GEP 1 to GEP 4.

c. Association of each cNMF program with canonical SCLC subtype signatures. Heatmap shows enrichment of representative SCLC-A, SCLC-N, SCLC-P and SCLC-I marker genes across GEP 1 to GEP 4. Note that GEP 3 showed the strongest enrichment for the SCLC-I-associated program, including Vim, Axl and Zeb2, whereas GEP 1 was enriched for SCLC-A features.

d. Comparison of cNMF program usage between Crebbp-KO and Crebbp-WT cells. Crebbp loss significantly altered usage of all four transcriptional programs, including increased usage of the SCLC-I-associated GEP 3 and decreased usage of GEP 2, consistent with a shift toward an SCLC-I-like transcriptional state. Box plots show the distribution of program usage across individual cells.

Supplementary Figure 14. Loss of *Crebbp* alters SCLC cell state through regulation of *Cdx2*.

a. Gene set enrichment analysis (GSEA) of snRNA-seq from *Crebbp*-WT and *Crebbp*-KO RP48 SCLC liver metastases. Heatmaps show normalized enrichment scores (KO vs. WT) across major cancer- and stroma-associated transcriptional programs. *Crebbp* loss leads to upregulation of MYC- and cell-cycle–associated signaling pathways.

b. Transcriptome-wide correlation between differential gene expression measured by bulk RNA-seq and snRNA-seq. A moderate Pearson correlation (r = 0.51) indicates substantial concordance between mRNA- and pre-mRNA based expression changes across platforms.

- **c.** Heatmap of bulk RNA-seq profiles for the top 500 differentially expressed genes from sorted *Crebbp*-WT and *Crebbp*-KO RP48 SCLC liver metastases. Clustering reveals the emergence of distinct transcriptional status upon *Crebbp* loss, including marked downregulation of *Cdx2* in *Crebbp*-KO cells.
- **d.** Pie charts summarizing differential H3K27ac ChIP-seq peaks identified in *Crebbp*-WT and *Crebbp*-KO cells.
- **e.** Doxycycline-induced CDX2 re-expression in *Crebbp*-KO RP48 cells suppresses proliferation and growth advantage in co-culture with *Crebbp*-WT cells. *Crebbp*-WT-TetON-CDX2 and *Crebbp*-KO-TetON-CDX2 RP48 cells were mixed at a 1:1 ratio and treated with 100 ng/mL doxycycline in co-culture for 0, 4, and 8 days. GFP/mCherry cell ratios were quantified by flow cytometry. n= 6 wells
- **f.** Doxycycline-induced CDX2 re-expression in *Crebbp*-KO RP48 cells suppresses transwell migration in co-culture with *Crebbp*-WT cells. *Crebbp*-WT-TetON-CDX2 and *Crebbp*-KO-TetON-CDX2 RP48 cells were mixed at a 1:1 ratio and treated with 100 ng/mL doxycycline for 4 days for transwell migration. GFP/mCherry cell ratios were quantified by flow cytometry. n= 6 wells
- **g.** *Crebbp*-mediated metastatic seeding suppression is CDX2-dependent. MOBA-seq analysis of genotype-specific metastatic seeding in control and CDX2-overexpressing RP48 cells that metastasized to the lung and liver. *Crebbp* inactivation increased metastatic seeding in control RP48 cells but had no effect in CDX2-overexpressing RP48 cells.
- **h.** *Crebbp*-mediated metastatic clonal expansion is CDX2-independent. MOBA-seq analysis of genotype-specific clonal expansion (90th percentile colony size) in control and CDX2-overexpressing RP48 cells that metastasized to the lung and liver. *Crebbp* inactivation increased metastatic clonal expansion in both control and CDX2-overexpressing RP48 cells.

Supplementary Figure 15. Loss of *Crebbp* alters liver metastatic microenvironment.

- **a.** Quality metrics of Xenium5k spatial transcriptomics data from *Crebbp*-WT and *Crebbp*-KO liver and lung metastases. Histograms show gene counts per cell.
- **b.** Spatial maps of ASCL1^{+} cells in *Crebbp*-WT and *Crebbp*-KO liver and lung metastases. *Crebbp*-KO lesions show a marked increase in ASCL1^{+} cell abundance in both liver and lung.
- **c.** UMAP visualization of spatially profiled cells from WT and KO liver (top) and lung (bottom) metastases. Color-coded clusters reveal extensive transcriptional reorganization in *Crebbp*-KO liver tumors, including pronounced remodeling of immune and stromal niches.
- **d.** Comparison of vascularized metastatic colony numbers between WT and KO liver metastases based on H&E staining. *Crebbp* loss significantly increases the number of vascularized metastatic colonies (Wilcoxon $p = 0.045$).
- **e.** Spatial mapping of glucose catabolism signatures across liver metastases. KO tumors show strong regional upregulation of glucose metabolic activity compared with WT. Right: Violin plots quantify signature scores for WT versus KO conditions.
- **f.** Spatial mapping of lipid metabolism signatures across liver metastases. KO tumors demonstrate increased lipid metabolic activity, suggesting metabolic rewiring following Crebbp inactivation. Right: Corresponding violin plots compare WT versus KO conditions.
- **g.** Representative image of *Crebbp*-KO liver metastases. Regions enriched for cancer-stroma interactions are highlighted.
- **h.** Representative spatial transcriptomic images showing (left) immune-cell recruitment pathways localized at the cancer–endothelial–immune interface and (right) T-cell exhaustion markers concentrated at the cancer–immune interface.
- **i.** Representative immunofluorescence of WT and KO liver metastases. KO tumors show increased infiltration of T cells (CD3), NK cells (NK1.1), and macrophages (F4/80).
- **j.** Quantification of CD3^{+} T cells, F4/80^{+} macrophages, and NK1.1^{+} natural killer cells in WT versus KO liver metastases. KO tumors exhibit significantly elevated infiltration across multiple immune subsets.
- **k.** Flow cytometry quantification of major immune subsets in WT versus KO liver metastases. *Crebbp* loss increases T cells, NK cells, but not B cells.

l. Analysis of human tumor mutation burden (TMB) from CREBBP-mutant versus CREBBP-wild-type cancers in the MSK-IMPACT cohort. CREBBP-mutant lesions display significantly increased TMB (Wilcoxon $p < 2.2 \times 10^{-16}$).

Supplementary Figure 16. *CREBBP* mutation is associated with improved survival specifically in patients treated with immune checkpoint inhibitors.
Multivariable Cox proportional hazards analysis of overall survival in the pan-cancer MSK-IMPACT cohort restricted to ICI-treated patients, including CREBBP mutation status, age, sex and metastatic status as covariates. *CREBBP*-mutant tumors were associated with significantly improved survival compared with CREBBP-wild-type tumors, whereas age, sex and metastatic status were not significantly associated with survival. These data identify *CREBBP* mutation as an independent correlate of favorable outcome among ICI-treated patients.
